# Cefepime-Induced Encephalopathy in Patients Treated for Urinary Tract Infection

**DOI:** 10.7759/cureus.65088

**Published:** 2024-07-22

**Authors:** Novera Inam, Nuzhat Nisa, Christopher Stewart, Mitchell Fisher, Suporn Sukpraprut-Braaten

**Affiliations:** 1 Family Medicine, Reid Health, Richmond, USA; 2 Medicine, Kansas City University of Medicine and Biosciences, Joplin, USA

**Keywords:** renal dysfunction, urinary tract infection treatment, neurotoxicity, cefepime, cefepime induced encephalopathy

## Abstract

Cefepime is a fourth-generation cephalosporin antibiotic administered intravenously used to treat various bacterial infections, including urinary tract infections. Administering cefepime to patients should be done with caution, understanding both potential risks and side effects. A 74-year-old female presented to the family medicine clinic with abdominal pain and a history of urinary tract infections. The workup included a CT scan that showed bowel obstruction and bladder wall thickening. Due to a history of urinary tract infections, three days following the presentation, the patient underwent an explorative laparotomy. Following the laparotomy, the patient was started on cefepime, a fourth-generation cephalosporin antibiotic. Five days following the initial presentation, the patient became confused and was nonverbal. An encephalopathy workup showed a negative MRI, but an EEG was consistent with encephalopathy. Cefepime was discontinued. Forty-eight hours after cefepime was discontinued, the patient returned to baseline with normal cognitive function. It is crucial that clinicians understand the different classifications of antibiotics, as well as the drugs and potential side effects of prescriptions. Cefepime can be used in gram-negative infections with resistance to more generic antibiotics. It has the ability to cross the blood-brain barrier, making it effective in treating meningitis. It has also been shown to cause encephalopathy as a side effect. It is important that clinicians understand the different generations of cephalosporins, as well as the cross-reactions and potential side effects of prescriptions. These factors must be considered when prescribing broad-spectrum antibiotics, such as cefepime.

## Introduction

Encephalopathy can be caused by many agents and is defined as a disease that affects the function or structure of the brain [1]. This can be temporary or long-lasting and varies greatly in presentation [2]. Common symptoms of encephalopathy include confusion, memory loss, behavior changes, and loss of consciousness [3]. We present a patient with encephalopathy, likely induced by cefepime, a fourth-generation cephalosporin antibiotic. There are five generations of cephalosporins [4]. As a fourth-generation cephalosporin, cefepime has the ability to penetrate the blood-brain barrier and attack bacteria in the CSF [4]. Due to its invasive nature and ability to attack diverse bacteria, cefepime is reserved for multi-drug-resistant bacterial infections and systemic infections [4]. While effective, encephalopathy has been reported in approximately 3% of patients taking cefepime, making it a high-risk antibiotic [2,5]. Additional risks associated with cefepime include allergic reactions, difficulty breathing, renal dysfunction, and diarrhea [6]. While antibiotics are generally safe, it is important to understand the risks and benefits associated with each medication prescribed.

## Case presentation

**Figure 1:**
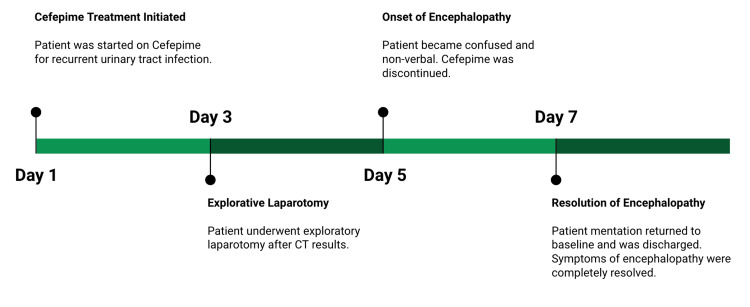
Timeline of the presented case

A 74-year-old female presented to the family medicine clinic with abdominal pain. The patient had a history of diabetes, asthma, hypertension, and recurrent urinary tract infections. A CT of the abdomen and pelvis was ordered. Findings from the CT included small bowel obstruction and chronic bladder wall thickening. The patient was alert and oriented on the day of admission with no symptoms of fever or hypertension. She was prescribed cefepime to eliminate the urinary tract infection and began taking it on day 1, the same day as the presentation. These measures were attempted but were not successful in eliminating her pain. Three days following the initial presentation, she underwent an exploratory laparotomy to eliminate the bowel obstruction. On day 5, she started to get confused. She could nod her head, but she was nonverbal. A detailed encephalopathy workup was performed. An MRI of the brain did not show signs of encephalopathy, such as abnormal signal intensity or edema. At this point, the differential diagnosis for the change in mentation included neurotoxicity from cefepime, infectious disease, and the pain management process. An EEG showed generalized slowing consistent with encephalopathy. Cefepime was discontinued, and a different antibiotic was initiated. She returned to baseline mentation 48 hours later, on day 7, from the initial presentation. A timeline of this case can be seen in Figure 1.

## Discussion

Key findings from this case report include the occurrence and resolution of probable cefepime-induced encephalopathy. Cefepime is frequently used to treat severe and complex urinary tract and abdominal infections, similar to the case presented here. Common side effects of cefepime in adults include diarrhea and rash; however, encephalopathy is a rare documented adverse effect [7-9]. In this patient, encephalopathy was drug-induced and manifested as confusion and a nonverbal state. Risk factors for developing encephalopathy with cefepime use include renal dysfunction, excessive dosing, and preexisting brain injury [10]. Notably, no additional treatment was required, highlighting the importance of drug cessation in resolving symptoms. Although specific testing to confirm cefepime as the cause was not conducted, the correlation between stopping the drug and symptom resolution within two days strongly supports this diagnosis.

Cefepime-induced encephalopathy has been reported at an increased rate over the last 10 years [11]. The mean age for this type of encephalopathy is 67, and 87% of patients report renal dysfunction [12]. Encephalopathy typically occurs five to 10 days following the initial administration of cefepime [9]. This case presented similarly to reported cases in the literature, but with the absence of renal failure. This underscores the need for vigilance regarding neurotoxic side effects in patients receiving cefepime, even though such occurrences are uncommon.

Cefepime and most cephalosporins are primarily excreted via the renal system; thus, patients with renal dysfunction must receive dosage adjustments [4,7]. Renal failure prolongs the half-life of cephalosporins in the blood and can induce neurotoxicity [7]. Crossing the blood-brain barrier is a relatively unique ability of cefepime, which makes it a good choice for certain meningitis infections [13]. The neurotoxicity of cefepime, along with other penicillins and cephalosporins, is not fully understood, but the predominant theory is that it inhibits the gamma-aminobutyric acid (GABA) system [14,15]. Inhibition of the GABA system leads to increased excitatory activity and subsequent cell injury, leading to encephalopathy [16]. The reduced threshold of excitation due to the downregulation of the GABA system is likely related to the structure of cephalosporins, although more research is being conducted [14,15].

The differential diagnosis for acute encephalopathy is broad but includes subdural hematomas, infectious agents (both systemic and neurologic), inflammatory conditions, and drug-related toxicity [1]. The history and physical examination in this case revealed a negative MRI for any hematoma, while the EEG showed signs consistent with encephalopathy. The patient presented here has a history of recurrent urinary tract infections and was taking cefepime to eliminate the infection. Infectious agents could contribute to the neurotoxic state; however, due to the quick resolution of symptoms following cessation of the drug, we concluded that CNS symptoms were due to cefepime toxicity. Thus, this case report describes an example of an acute drug-related neurotoxic form of encephalopathy.

The limitations of this study include the diagnosis of encephalopathy with no signs on the MRI. While little research has been done in identifying the sensitivity and specificity of drug-induced encephalopathy, the accuracy of using MRI to identify Wernicke’s encephalopathy, encephalitis, and autoimmune encephalopathy is well below 100% [17,18]. This indicates that an MRI without symptoms of encephalopathy does not rule out a diagnosis. An additional limitation is that no specific diagnostic tests were performed to confirm the drug’s causative role. Although this was the case, the concurrence of drug discontinuation with the subsequent abatement of symptomatology and EEG findings supports the diagnosis of cefepime-induced encephalopathy. Lastly, the lack of long-term follow-up data restricts our understanding of the potential recurrence or long-term consequences of the encephalopathy. Despite these limitations, this case highlights the importance of recognizing the neurotoxic side effects of cefepime. Further reporting of cefepime-induced encephalopathy and neurotoxicities is needed to further understand the potential adverse side effects of cefepime and potential contraindications.

## Conclusions

Cefepime-induced encephalopathy is a rare but significant medical occurrence. While the majority of patients with encephalopathy present with risk factors such as renal dysfunction or previous brain injury, this report documents a patient with cefepime-induced encephalopathy without these risk factors. This atypical presentation underscores the importance of recognizing drug-induced complications even in patients without traditional risk factors. Patients suspected of having cefepime-related neurotoxicity should alter their antibiotic treatment and be further evaluated. Reporting these cases is important for enhancing awareness among clinicians and improving patient outcomes.
